# Prognostic biomarkers in oral squamous cell carcinoma: current evidence and future directions

**DOI:** 10.3389/fonc.2025.1692294

**Published:** 2025-12-05

**Authors:** Phillipp Brockmeyer

**Affiliations:** Department of Oral and Maxillofacial Surgery, University Medical Center Goettingen, Goettingen, Germany

**Keywords:** oral squamous cell carcinoma, OSCC, prognostic factors, malignancy, progression, risk stratification

## Abstract

Oral squamous cell carcinoma (OSCC) exhibits significant prognostic heterogeneity. This has prompted extensive research into biomarkers that can predict clinical outcomes beyond conventional staging systems. This mini review summarizes findings from the existing literature to provide a comprehensive examination of the prognostic significance of malignancy and progression factors in OSCC, offering insights into future perspectives. There is clear evidence that molecular and protein-based biomarkers, in addition to established clinical and histopathological features, such as lymph node involvement, extranodal spread, and depth of invasion, strongly correlate with overall survival (OS), disease-free survival (DFS), and progression-free survival (PFS). Integrating different gene expression signatures, microRNA (miRNA) profiles, and changes in intracellular signaling pathways enables more differentiated risk stratification. Protein biomarkers such as cyclin D1, trophoblast cell surface antigen 2 (TROP2), urokinase-type plasminogen activator receptor (uPAR), and E-cadherin have been shown to provide clinically useful prognostic information. These results underscore the importance of incorporating biomarkers into individualized risk stratification to enhance personalized treatment regimens and outcomes for OSCC patients. Currently, however, established clinical and histopathological parameters, as well as a limited number of validated molecular profiles, remain the most reliable prognostic indicators. While identifying new biomarkers is promising, establishing standardized protocols and implementing careful prospective validation are essential to ensuring their seamless integration into standard clinical practice.

## 1 Introduction

Oral squamous cell carcinoma (OSCC) poses a substantial global health concern (1), marked by heterogeneous clinical outcomes that defy comprehensive prediction by the prevailing TNM staging system (2). The prevailing risk stratification models are predominantly dependent on clinical and histopathological parameters, encompassing lymph node involvement, depth of invasion, and metastatic spread (3). While these features are clinically robust and widely applied, they insufficiently capture the biological heterogeneity of OSCC and often fail to reflect the variability in individual patient trajectories (4). This prognostic heterogeneity has driven extensive research investigating biological factors that influence tumor progression and metastasis (5). The goal of such research is to inform clinical decision-making and optimize patient care (5). Recent advances in genomic and proteomic technologies have enabled the discovery of a diverse spectrum of candidate biomarkers, including different protein expression profiles (6–21), molecular and genetic alterations (22, 23), dysregulated signaling pathways (24), immune landscape characteristics (25–28), and distinct microRNA (miRNA) expression signatures (29, 30). Collectively, this expanding repertoire of biomarkers presents considerable opportunities for refining prognostication and guiding therapeutic strategies; however, it also underscores key challenges, particularly the need for rigorous validation across heterogeneous patient cohorts and for elucidation of the interplay among multiple biomarkers in shaping clinical outcomes.

This mini review synthesizes evidence from the extant literature to evaluate the prognostic utility of malignancy and progression factors in the biology of OSCC. These studies encompass systematic reviews, meta-analyses, and primary research investigations that collectively provide a comprehensive perspective on how biological markers can help to identify patients with high risk.

## 2 Methods

A search of the PubMed and ScienceDirect databases was carried out using the following MeSH (medical subject headings) terms and keywords in various combinations: “oral squamous cell carcinoma”, “OSCC”, “oral cancer”, “biomarker”, “prognostic factors”, “molecular markers”, “gene expression”, “protein marker”, “miRNA”, “immunohistochemistry”, “survival”, “prognosis”, “overall survival”, “disease-free survival”, “progression-free survival”, “mortality”, and “outcome”.

## 3 Established clinical and histopathological features

The clinical and histopathological assessment is a critical component of the diagnostic process for OSCC, as it provides fundamental prognostic information that is essential for informed treatment planning and patient management. A comprehensive systematic review and meta-analysis by Gonzalez-Ruiz et al. encompassing 63,114 patients from 255 studies demonstrated that nodal involvement, extranodal extension, high depth of invasion, and distant metastasis were associated with hazard ratios (HR) for overall survival (OS) between 2.04-5.19 and for disease-free survival (DFS) between 1.97-2.78 (3). In a similar vein, da Silva Dolens et al. conducted a comprehensive analysis of 172 studies, revealing that a high depth of invasion, extranodal extension, perineural and lymphovascular invasion, positive surgical margins, bone invasion, high tumor thickness, pattern of invasion, tumor budding, and tumor-stroma ratio exhibited significant associations with diminished survival (OS HR: 1.09–2.96; DFS HR: 1.53–2.82) (31).

Adeoye et al. further corroborated these findings in their retrospective cohort of 467 patients, demonstrating that perineural and lymphovascular invasion, bone invasion, and extranodal extension predicted poor OS, DFS, and progression-free survival (PFS) (32). The prognostic significance of invasion patterns was further substantiated by Welkoborsky et al., who evaluated proliferating cell nuclear antigen (PCNA), mindbomb E3 ubiquitin protein ligase 1 (MIB1), protein 53 (p53), non-metastatic protein 23 (nm23), and DNA analysis in 42 T1N0M0 OSCC specimens, finding associations with disease-specific survival (DSS) (33).

Although meta-analyses support the prognostic value of clinical and histopathological parameters in OSCC, limitations remain regarding standardization and clinical applicability. Considerable heterogeneity in parameter assessment (OS HR: 1.09–2.96) raises concerns about reproducibility and interobserver reliability (31, 34). Additionally, discrepancies in clinical and pathological staging (up to 25% of cases) and prognostic variability within the same TNM stages highlight the inadequacies of current risk stratification (35, 36).

## 4 Molecular and genetic signatures

Molecular profiling has revealed numerous biomarkers with prognostic significance in OSCC. Lohavanich et al. conducted a prospective cohort study with 270 tissue samples and identified 20 gene probe sets associated with reduced PFS (HR: 2.7 and 1.6) (22). Subsequent exploration of these signatures was conducted by Mendez et al., who identified LAMC2 in a 131-gene signature associated with poor OSCC-specific survival (HR: 5.43) in 119 patients (23). The authors concluded that the combination of gene expression and tumor stage provides a more accurate survival prediction for OSCC patients than the tumor stage alone (23).

The advent of miRNA biomarkers has led to their emergence as potent prognostic indicators. Troiano et al. conducted a systematic review and meta-analysis of 1,200 OSCC samples and found that 16 miRNAs (9 upregulated, 7 downregulated) predicted survival outcomes (OS HR: 2.65; DFS HR: 1.95) (29). Furthermore, Yoon et al. identified miRNAs such as miR-375 and miR-214-3p as potential predictors of DFS in early stage OSCC (30).

The analysis of pathway alterations offers an additional dimension to molecular prognostication. Fan et al. evaluated mutational signatures and pathway alterations in 165 advanced staged OSCC patients, finding that NOTCH, RTK/RAS/MAPK, and TGF-β pathway alterations predicted worse OS and DFS (24). An investigation into the subject was conducted by Shen et al., who identified seven CpG methylation sites capable of predicting OS (HR: 2.79-3.69) in 313 OSCC patients (37).

Cell cycle regulators represent another significant category of molecular biomarkers. Ramos-García et al. conducted a systematic review and meta-analysis of 2,942 patients and found cyclin D1 overexpression associated with worse OS (HR: 2.00) and DFS (HR: 1.46) (38). Piao et al. identified ubiquitin-specific peptidase 22 (USP22) as a novel prognostic marker in 319 patients, with overexpressing this protein predicting poor OS and DFS (39).

In their study, Mohanta et al. investigated the role of cancer stem cell genes in a cohort of 313 OSCC patients and 28 validation cases (40). Their findings revealed that the expression of cyclin-dependent kinase 1 (CDK1) and NAD(P)H quinone dehydrogenase 1 can serve as a prognostic marker, indicating a high probability of poor DFS and OS (40). Zhao et al. identified a three-mRNA signature comprising plasminogen activator urokinase (PLAU), claudin 8 (CLDN8), and cyclin-dependent kinase inhibitor 2A (CDKN2A) that predicted OS (41).

Although studies have provided substantial evidence for prognostic molecular and genetic biomarkers in OSCC, their clinical applicability and reproducibility remain limited. Reported hazard ratios (HR: 1.46–5.43) are confounded by gene signature heterogeneity and poor overlap across cohorts (42). Small sample sizes, a lack of standardized assessment protocols, and an absence of independent validation cohorts further hinder implementation (43, 44). Additionally, focusing on single-layer molecular analyses without integrating multi-omics or tumor microenvironmental (TME) factors restricts predictive accuracy. Standardized validation frameworks are therefore essential before clinical adoption (4, 45).

## 5 Protein-based biomarkers

Protein-based biomarkers have been demonstrated to offer clinically actionable prognostic information in cases of OSCC. Fong et al. (6) and Tang et al. (7) identified trophoblast cell surface antigen 2 (TROP2) as a significant predictor of poor OS, with Fong et al. reporting a relative risk of 2.26 in 90 patients (6). Cell adhesion molecules have demonstrated particular prognostic value, as evidenced by a systematic review and meta-analysis of 2,553 patients by Lorenzo-Pouso et al. (8). This analysis revealed that E-cadherin expression was associated with a favorable prognosis (OS HR: 0.41; DFS HR: 0.47) (8).

The urokinase plasminogen activator system has been the subject of extensive research. Christensen et al. evaluated urokinase plasminogen activator receptor (uPAR), tissue factor (TF), and epidermal growth factor receptor (EGFR) in 191 patients, finding that uPAR expression was predictive of poor OS (HR: 1.595) (9). Dos Santos et al. identified hypoxia-inducible factor 1 alpha (HIF1-alpha) as a predictor of local DFS and OS in 66 patients (46).

Furthermore, the prognostic value of cytoskeletal proteins has been demonstrated. Coelho et al. discovered that the expression of keratins 17 and 19 was associated with a fourfold increase in the risk of mortality and relapse (10). In a study, Lo Muzio et al. identified heat shock protein 27 (HSP27) as an independent prognostic marker (OR: 4.404) in a cohort of 57 patients with OSCC (11).

Another promising category is represented by stem cell markers. Oliveira et al. investigated CD44 and CD24 immunophenotypes in 157 patients, finding associations with poor OS (12). In a n other study, Yoshihama et al. evaluated the expression levels of SOX2, Oct4, c-Myc, KLF4, and brachyury in a cohort of 108 patients (13). Their findings revealed that low co-expression of SOX2, KLF4, and brachyury expression was associated with reduced DSS and DFS rates (13).

Proliferation markers have been the subject of extensive research. Montebugnoli et al. conducted prospective studies evaluating Ki-67 in distant mucosa (47 and 42 patients, respectively), finding it to be a predictive factor of DFS in early-stage OSCC (14, 15). Monteiro et al. evaluated a series of biomarkers, including but not limited to EGFR, p53, p16, p27, cyclin D1, cyclin A2, cyclooxygenase-2, Ki-67, Bcl-2, and vascular endothelial growth factor receptors (16, 17). This study was conducted on a cohort of 67 patients, and the researchers developed immunohistochemical scores that were capable of predicting survival outcomes (17).

Harris et al. employed proteomic analysis of 43 OSCC specimens and identified desmoplakin, plakophilin-1, tripartite motif-containing protein 29 (Trim29), S100A8, and S100A9 as associated with disease-specific death, metastasis, and recurrence (18). Galvis et al. investigated 13 proteins associated with the cell cycle and invasion in 132 patients with OSCC, finding different expression patterns that predicted outcomes (19).

In a recent study, Ramasubramanian et al. identified a novel biomarker, TBRG4, which was found to promote progression and predict poor OS in a cohort of 51 patients (20). Shin et al. (21) and Yoshihama et al. (13) investigated KiSS-1 in OSCC. Shin et al. found KiSS-1 to be a predictor of poor OS and DFS (21).

Protein biomarker studies in OSCC indicate prognostic value; however, methodological limitations restrict clinical applicability. Concerns about reproducibility and validation arise from small sample sizes, heterogeneous study designs, and non-standardized immunohistochemical protocols and scoring systems (47–49). The lack of independent validation cohorts, together with challenges in antibody specificity and immunohistochemical standardization, further hinders translation (43, 50, 51). The predominantly single-center, retrospective nature of these studies, lacking multi-institutional frameworks, limits their generalizability. This underscores the need for standardized protocols and robust external validation before clinical use (50, 52, 53).

## 6 Immune microenvironment and viral biomarkers

The tumor immune microenvironment has emerged as a critical determinant of prognosis. Feng et al. conducted a multiparametric immune profiling study on 119 HPV-negative OSCC patients, evaluating CD3+, CD8+, FoxP3+, CD163+, PD-L1+, and antigen processing machinery components (25). Their findings revealed specific immune patterns that were predictive of OS (25). Moratin et al. expanded on this in 222 patients, demonstrating that programmed death-ligand 1 (PD-L1), EGFR cyclooxygenase-2, CD8, and natural killer cell markers predicted OS and PFS (26).

The human papillomavirus (HPV) status has been investigated as a prognostic factor, particularly in relation to other biomarkers. Zhao et al. confirmed HPV status as an independent predictor in OSCC (27). In another study, Loeschke et al. evaluated the presence of HPV, p16, and HMGA2 in a cohort of 91 patients (28). Their findings revealed that HMGA2 expression was a significant predictor of both OS and DFS, with (28).

Studies examining the tumor immune microenvironment and HPV status in OSCC have critical limitations that compromise their clinical applicability. The main issue is the low prevalence of HPV in true oral cavity cancers, which is well-documented at 2.2%-6%. Most studies show that HPV has no prognostic impact. This contrasts sharply with oropharyngeal cancers (54, 55). Contradictory findings about whether p16 positivity predicts favorable outcomes raise concerns about potential anatomical misclassification, particularly the inclusion of base-of-tongue tumors. These tumors should be classified as oropharyngeal rather than oral cavity cancers (55, 56). Additionally, small sample sizes and a lack of standardized immune profiling protocols limit the reproducibility and generalizability of these findings (50, 57).

## 7 Novel diagnostic approaches and composite scores

Innovative diagnostic approaches in OSCC show promise, yet they face significant barriers to translation. Ishikawa et al. investigated salivary metabolomics in 72 patients with OSCC (58). Their findings identified 3-methylhistidine concentration as potential biomarkers for OS and DFS (58). The study reported an HR of 4.865 and a p-value of 0.012, indicating the significance of these findings (58). Clinical implementation remains premature given the study’s small sample size, lack of external validation, and broader salivary-biomarker challenges—interindividual variability, diurnal fluctuations, and non-standardized collection—all of which require resolution (59–61). Chien et al. conducted a study in which they evaluated the expression of osteopontin in a cohort of 122 patients (62). Their findings indicated that osteopontin exhibited marginal independent prognostic value for OS (62).

A number of studies have developed composite scores with the objective of improving prognostication. Monteiro et al. developed an immunohistochemical score incorporating multiple biomarkers to predict outcomes (17). Trivedi et al. conducted a site-specific analysis of prognostic biomarkers in 135 buccal and tongue carcinomas (63). The analysis evaluated the presence of EGFR, signal transducers and activators of transcription 3 (STAT3), human epidermal growth factor receptor 2 (H-ras), c-myc, p53, cyclin D1, p16, retinoblastoma protein, and Bcl-2 in order to assess recurrence-free and overall survival (63). Monteiro et al. and Trivedi et al.’s composite scoring systems also lack validation across diverse populations and remain constrained by challenges in immunohistochemical standardization (49).

**Figure 1:**
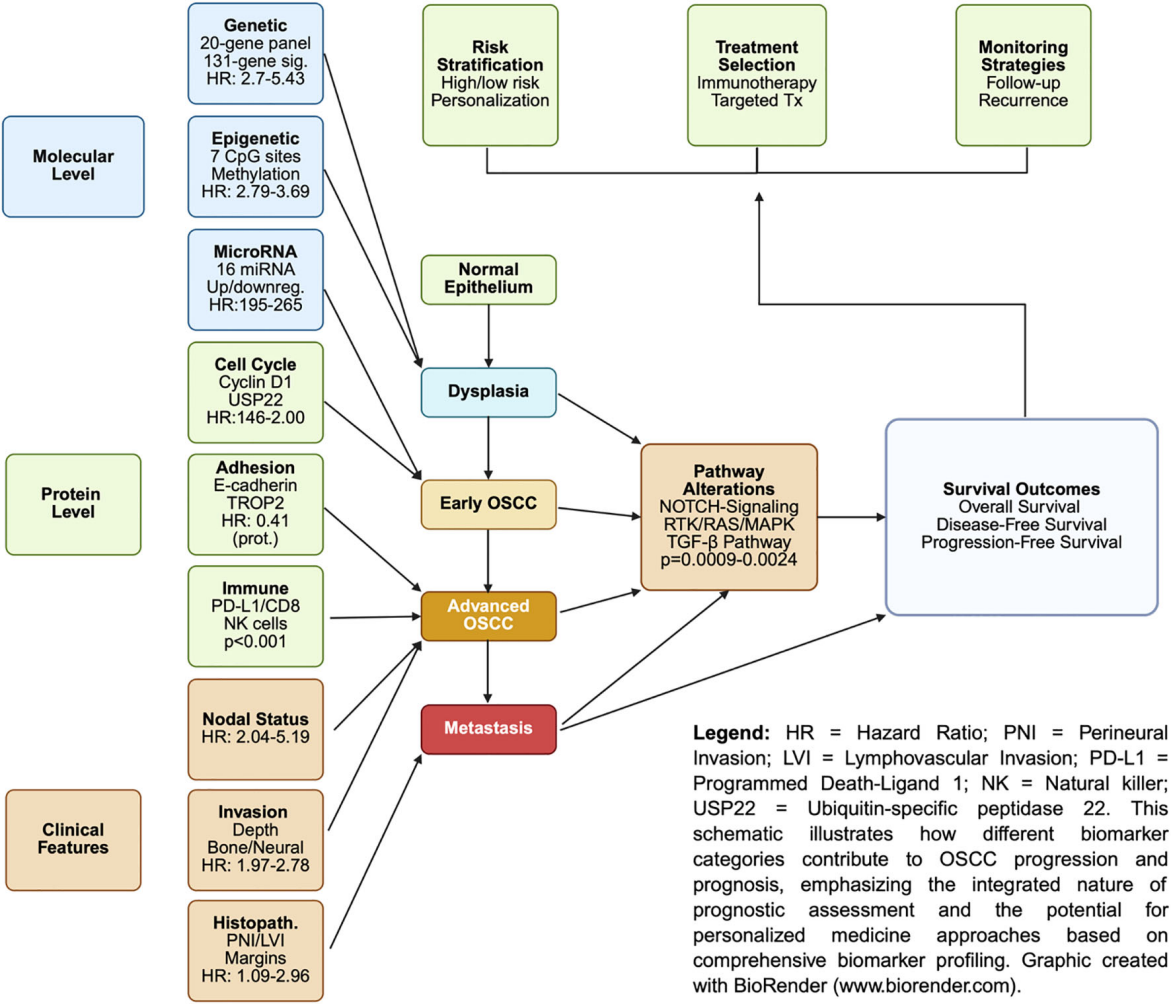
Schematic overview of biomarker integration in OSCC progression and prognosis.

**Table 1** summarizes the main clinicopathological and molecular biomarkers associated with OS, DFS, and PFS in OSCC. **Figure 1** shows a graphical representation of the integration of biomarkers in OSCC progression and prognosis.

**Table 1:** Major prognostic biomarkers in oral squamous cell carcinoma.

Category	Specific marker	Cohort size	Effect size	Outcome	Reference
Clinicopathological	Nodal involvement	63,114	HR: 2.04-5.19	OS	González-Ruiz et al., 2025 (3)
	Invasion depth	63,114	HR: 1.97-2.78	DFS	González-Ruiz et al., 2025 (3)
	Histopathological features*	172 studies	HR: 1.09-2.96	OS/DFS	da Silva Dolens et al., 2021 (31)
Gene Expression	20-gene panel	349	HR: 2.7 (2.0-3.8)	PFS	Lohavanichbutr et al., 2012 (22)
	131-gene signature	119	HR: 5.43	CSS	Mendez et al., 2009 (23)
	Pathway alterations†	165	p=0.0024/0.0009	OS/DFS	Fan et al., 2021 (24)
Epigenetic	7 CpG methylation sites	313	HR: 2.79-3.69	OS	Shen et al., 2017 (37)
MicroRNAs	16 miRNA panel	1,200	HR: 2.65 (2.07-3.39)	OS	Troiano et al., 2018 (29)
			HR: 1.95 (1.28-2.98)	DFS	
Protein Markers	Cyclin D1	2,942	HR: 2.00	OS	Ramos-García et al., 2018 (38)
			HR: 1.46	DFS	
	E-cadherin	2,553	HR: 0.41‡	OS	Lorenzo-Pouso et al., 2023 (8)
			HR: 0.47‡	DFS	
	TROP2	90	RR: 2.26 (1.33-3.83)	OS	Fong et al., 2008 (6)
	USP22	319	p<0.001	OS/DFS	Piao et al., 2012 (39)
	Heat shock protein 27	57	OR: 4.404	OS	Lo Muzio et al., 2006 (11)
	uPAR	191	HR: 1.595	OS	Christensen et al., 2017 (9)
Immune Markers	PD-L1/EGFR/CD8/NK	222	p<0.001	OS/PFS	Moratin et al., 2021 (26)
	Immune signatures	119	p<0.05	OS	Feng et al., 2017 (25)
Metabolomic	3-methylhistidine	72	HR: 4.865	OS	Ishikawa et al., 2022 (58)
Cancer Stem Cells	54 CSC genes	313	p<0.05	OS/DFS	Mohanta et al., 2019 (40)

OS, Overall Survival; DFS, Disease-Free Survival; PFS, Progression-Free Survival; CSS, Cancer-Specific Survival; HR, Hazard Ratio; OR, Odds Ratio; RR, Relative Risk; CI, Confidence Interval; USP22, Ubiquitin-specific peptidase 22; uPAR, Urokinase plasminogen activator receptor; PD-L1, Programmed death-ligand 1; EGFR, Epidermal growth factor receptor; NK, Natural killer cells; CSC, Cancer stem cell. *Includes depth of invasion, extranodal extension, perineural invasion, lymphovascular invasion, margins, bone invasion, tumor thickness, pattern of invasion, tumor budding, tumor-stroma ratio †NOTCH, RTK/RAS/MAPK, TGF-beta pathways ‡Protective effect (lower hazard ratio indicates better survival).

## 8 Discussion and future directions

Current evidence suggests that biomarkers may help identify patients with high-risk OSCC (5). Adverse biomarkers are associated with HRs between 1.5–5.0, whereas favorable markers, such as high E-cadherin expression (8), are linked to HRs of about 0.4–0.5.

However, conventional clinicopathological variables, such as nodal involvement, extranodal extension, and depth of invasion, are still the most informative predictors of poor OS and DFS (3). Additional histopathological features, such as perineural and lymphovascular invasion, positive margins, and bone infiltration, complement these variables for risk stratification (31), and the accumulation of multiple adverse factors accelerates disease progression (3, 31, 32). Nevertheless, these parameters alone do not capture the biological heterogeneity of OSCC. This underscores the need for standardized biomarker assays with validated cutoff values, reproducible analytical methods (4), and multi-marker panels that integrate histopathological, molecular, and proteomic data. These improvements would enhance prognostic accuracy (64).

Much of the existing evidence derives from retrospective analyses; therefore, prospective multicenter validation and randomized trials of biomarker-guided management are necessary to establish clinical utility and cost-effectiveness. Individual biomarker classes, particularly gene expression panels (22), protein markers (38), and methylation signatures (37), have already demonstrated prognostic value. However, integrated multi-omics approaches are likely to yield synergistic signatures that outperform single-layer models (65). In this context, artificial intelligence (AI) and machine learning methods can combine clinical and pathological features, molecular signatures, and digital histopathology to generate robust, individualized risk estimates and uncover morphomolecular patterns imperceptible to humans (66).

Prognostic factors can help to guide therapy modulation, especially in the era of immunotherapy (45). The TME is pivotal for both prognosis and treatment response (67). Immunologic markers, such as PD-L1, CD8+ T cells, NK-cell signatures, and composite immune scores, may identify candidates for checkpoint inhibition (26). Conversely, tumors enriched with immunosuppressive cancer-associated fibroblasts (CAF) may require priming regimens or CAF-targeted combinations, including TGF-β inhibition or fibroblast reprogramming (68). Loss of E-cadherin during epithelial-mesenchymal transition (EMT) and metastasis appears to facilitate immune evasion (8). Meanwhile, alterations in the NOTCH, RTK/RAS/MAPK, and TGF-β pathways converge on immune regulation and fibroblast activation, linking prognostic biology to therapeutic vulnerability (24). High-risk miRNA and methylation profiles warrant intensified surveillance (29, 37), and circulating biomarkers enable the early detection of recurrence, as well as the dynamic reassessment of risk (58).

Validated biomarker profiles have the potential to refine patient stratification and guide therapeutic decisions across the entire continuum of care (69). It is crucial to integrate these profiles into the current TNM staging system, as TNM only captures the anatomical extent of disease, not its biological heterogeneity (70). This heterogeneity contributes to divergent outcomes among patients within the same stage (35, 36). Molecular signatures (22), signaling pathway alterations (24), and protein markers (38) could complement TNM staging by providing critical information on tumor aggressiveness, metastatic potential, and treatment resistance (22). High-risk constellations may justify treatment intensification, including adjuvant systemic therapy, extended radiation fields, or multimodal strategies. Conversely, favorable profiles (8) in the context of low-risk clinical features may support treatment de-escalation. A pragmatic approach would first apply the TNM classification, followed by a complementary risk assessment using validated biomarker panels. Ideally, a molecular tumor board would review and interpret the integrated findings.

## 9 Conclusions

The biological determinants of OSCC offer prognostic insights that surpass anatomical staging. Integrating clinicopathological parameters with molecular and protein biomarkers improves risk stratification and supports rational treatment escalation or de-escalation. This integration also enables personalized care. Priorities include standardizing protocols, integrating multi-omics, developing and validating AI-based predictive models, and conducting robust, multicenter, prospective studies, including randomized, biomarker-based trials. The overarching goal is to routinely incorporate validated biomarker panels into TNM staging to achieve an individualized, actionable risk assessment.
